# The Story of the Insane from Year to Year

**Published:** 1891-09-05

**Authors:** 


### The Story of the Insane from Year to Year.

(Fourth Series.)

[Annual Reports, Pamphlets, &c., should be addressed to The Editor, The Lodge, Porchester Square, London, W.]

### THE COST OF ASYLUM BUILDINGS.

"Enquirer" writes as follows: In your paper of August 15th, "English Lunacy in 1890," you state "a very good asylum can be built for £130 a bed," and this after reference to the cost to the ratepayers of London on the boarding-out system. If an asylum can be built for £130 a bed, I, as a London ratepayer, would like to ask why the Council are spending on their new asylum at Claybury £200 a bed? I understand this asylum is to contain 2,000 beds and that more than £400,000 is being laid out.

I agree with you that with economy £130 a bed will build an asylum, and some built a few years back, I believe for the Metropolitan Asylums Board, cost something like £80 a bed. I daresay these would not be considered quite up to date for an acute asylum, but there is a long margin between £80 and £200.

### NORWICH BOROUGH ASYLUM.

This asylum contains 259 patients. During the year 1890 it admitted 77. There were 29 recoveries, and 24 deaths; the percentage of the former being 38·15, and of the latter 8 63. The influenza paid the asylum a visit, and we learn that every official in the asylum, with a solitary exception, suffured from the epidemic. Many patients were stricken, but there was no fatal case.

Dr. Harris thinks the time is coming when it will be deemed wise to provide workshops for women, and he adds that the census will show the necessity for the step especially in manufacturing districts; but, continues Dr. Harris, "no census is necessary to prove the oft-told tale Satan finds some mischief still for idle hands to do, and I believe idleness begets idleness, and idleness destructiveness, and so on to the loss of hope, and then a patient may become a chronic lunatic, and a life-long expense." No doubt, no doubt! But how is this Satanic influence to be counteracted in asylums, and how are we to get the refractory women to employ themselves either in workshops or elsewhere? We are not quite sure about the life-long expense of the chronic lunatic being greater than the ultimate cost of his early cure. Has Dr. Harris never reckoned up the number of his "recoveries" who have gone into the world and propagated an insane family, many of whom will fall on the rates and vastly increase the national burden? So far as the "expense" goes it would be better far if he cured nobody.

The New Lunacy Bill is noticed as follows: "I regret to say, under the New Lunacy Bill, my duties of certifying will be more than doubled, and apparently without any benefit to the insane or public at large." The enactions of of this unfortunate Bill are irksome enough with Dr. Harris 250 patients; but let him imagine what they mean where there are 2,000 cases to deal with. But we must live in hope; we have nothing left but hope. We believe there are upwards of six hundred members in our House of Commons, and it will be strange if not one of these has the courage to bring in a new Bill before long.

The visitors say, "Dr. Harris has for many years merited our greatest confidence and highest esteem."

The balance in favour of the farm is £68, but we see nothing down for rent, patients' labour, or interest on capital, and the prices charged to the asylum for provisions are not given.

The weekly charge to the Unions is 9s. 4d.

### KENT COUNTY ASYLUM AT CANTERBURY.

The patients in this asylum increased during the year from 795 to 844. There were 171 admissions; 40 recoveries and 59 deaths. The percentage of recoveries on the admissions was 28, and of the deaths on the average number resident, 7. Of the admissions we find that 22 owed their insanity to drink, and 42 to hereditary influence; 23 of the deaths were from brain disease, and 7 from phthisis. The low death-rate shows that the general health of the patients was above the average in asylums. Influenza visited the asylum, but only four patients were attacked, the majority of those seized being officials of the institution. The Visiting Committee do not print their report. The Commissioners in Lunacy recommend additions to the asylum for 200 patients. We learn from the Commissioners' report that the weekly charge to the Kent unions is 8s. 2d. per head; but no financial statements are elsewhere given.

### THE LEICESTERSHIRE COUNTY ASYLUM.

Four hundred and sixty-nine patients were in residence on New Year's day, 1890, and on the last day of the year there were 471, showing that the numbers were more nearly stationary than in most asylums. The admissions were 111; the recoveries 38, and the deaths 58. The recoveries are 36 19 per cent. on the admissions, and the deaths 12 2 on the average number resident. Intemperance in drink is the assigned cause of the disease in fifteen instances, and hereditary influence in seventeen. Nine of the deaths were from phthisis and five from pneumonia and other lung diseases. Dr. Higgins draws "attention to the unfavourable nature of a large proportion of the admissions of last year as regards recovery or improvement; 48 of the 109 admissions were between 50 and 60 years of age; 25 were upwards of 00; and in 22 instances the cases must be regarded as hopeless, since the duration of their insanity before admission was given in their certificates as upwards of twelve months. This is an unfavourable material from which to effect cures." So it is and so it will continue to be, and when Dr. Higgins can keep his percentage of recoveries up to an average of 44, which he seems to have done during the last seven years, he may take a little credit to himself and feel satisfied he has not done badly. The farm accounts show a profit of £335, but the balance sheet, like that of so many other asylums, says nothing of rent of land, interest on capital, patients' labour, nor the prices at which farm produce is supplied to the house. The weekly cost of maintenance is 9s. 2¼d. per head.

